# Immunotherapy for Parkinson’s disease

**DOI:** 10.1016/j.nbd.2020.104760

**Published:** 2020-01-21

**Authors:** Aaron D. Schwab, Mackenzie J. Thurston, Jatin Machhi, Katherine E. Olson, Krista L. Namminga, Howard E. Gendelman, R. Lee Mosley

**Affiliations:** Department of Pharmacology and Experimental Neuroscience, Center for Neurodegenerative Disorders, University of Nebraska Medical Center, Omaha, NE 68198-5110, United States of America

**Keywords:** Immune transformation, Regulatory T cells, Tregs, Effector T cells, Teffs, Granulocyte-macrophage colony stimulating, factor, Immune homeostasis, Neurodegeneration, Neuroprotection, Nigrostriatal degeneration, Neuroinflammation, Parkinson’s disease, Neurodegenerative disorders, Alzheimer’s disease, Ischemic stroke, Traumatic brain injury

## Abstract

With the increasing prevalence of Parkinson’s disease (PD), there is an immediate need to interdict disease signs and symptoms. In recent years this need was met through therapeutic approaches focused on regenerative stem cell replacement and alpha-synuclein clearance. However, neither have shown long-term clinical benefit. A novel therapeutic approach designed to affect disease is focused on transforming the brain’s immune microenvironment. As disordered innate and adaptive immune functions are primary components of neurodegenerative disease pathogenesis, this has emerged as a clear opportunity for therapeutic development. Interventions that immunologically restore the brain’s homeostatic environment can lead to neuroprotective outcomes. These have recently been demonstrated in both laboratory and early clinical investigations. To these ends, efforts to increase the numbers and function of regulatory T cells over dominant effector cells that exacerbate systemic inflammation and neurodegeneration have emerged as a primary research focus. These therapeutics show broad promise in affecting disease outcomes beyond PD, such as for Alzheimer’s disease, stroke and traumatic brain injuries, which share common neurodegenerative disease processes.

## 1. Introduction

Parkinson’s disease (PD) is the most common movement disorder and second in prevalence only to Alzheimer’s disease (AD) as the most common neurodegenerative disorder. Ninety percent of cases are sporadic (Olanow et al., 2009; Tysnes and Storstein, 2017; Schulze et al., 2018). Clinically, PD manifests with resting tremors, mask-like faces, bradykinesia, shuffling gait, and rigidity. Most clinical signs and symptoms are linked to the loss of the neurotransmitter dopamine with progressive degeneration of dopaminergic neurons in the substantia nigra pars compacta (SNc) and loss of their efferent presynaptic termini in the striatum (Dauer and Przedborski, 2003; Kalia and Lang, 2015). The natural history of the disease is insidiously progressive, and thus by the time symptoms present, PD patients have lost 50% or more of the SNc dopaminergic neurons and up to 80% of the efferent termini (Surmeier et al., 2017). The incidence and prevalence of PD parallel advancing age and affects up to 1% of the population above 60 years of age (de Lau and Breteler, 2006). By 2016, PD prevalence had increased to 6.1 million people worldwide, up from 2.5 million people in 1990 (Dorsey et al., 2018a). This doubling in disease prevalence is attributable both to an increasing aged population (74% increase over the same period), but also due to reduced activities associated with neuroprotection, such as smoking, and increased exposure to industrial byproducts (Dorsey et al., 2018b). Even more disturbing is a predicted three-fold increase in PD to 17.5 million anticipated by 2040. With increased longevity and obvious continued environmental exposures affecting disease onset and progression, advancements in diagnosis and treatment are of immediate need.

While diminished dopamine is primarily due to lost pre-synaptic neurons, post-synaptic neurons also exhibit a general upregulation of dopamine receptors with corresponding increased sensitivities to dopaminergic ligands (Lee et al., 1978). Thus, current PD therapies primarily consist of dopamine replacement for neurotransmitter loss and dopamine agonists for upregulated dopamine receptors; both target disease symptoms, yet offer no curative outcomes. For curative outcomes, strategies that target replacement of lost or injured neurons through stem cell therapies, amelioration of neural injuries through growth factors, elimination of misfolded protein aggregates that contribute to neuronal injuries, or modulation of the diseased brain microenvironment will most likely be necessary (Jankovic and Aguilar, 2008). Underlying all these strategies are neuroinflammation and oxidative stress within the brain’s microenvironment that impede successful implementation. Indeed, neuroinflammation contributes to neurodegenerative processes and provides the driving force for much of the disease progression. Therefore, we posit that a positive outcome for any of these therapeutic pathways necessitates harnessing immune responses that are operative during ongoing disease.

Innate immune activation in PD is triggered by extracellular misfolded proteins released from dead or damaged neurons. These molecular aggregates incite microglial activation and the consequent release of pro-inflammatory immune factors that damage neighboring neurons and neuronal connections (Mosley et al., 2012). Adaptive immune responses by T cells or antibodies follow breaks or deviations in immune tolerance and the emergence of effector memory CD8+ or CD4+ T cells that can exacerbate microglial responses and neuronal vulnerability (Kannarkat et al., 2013; Allen Reish and Standaert, 2015). Recent research activities show direct links between both innate and adaptive immunity in PD pathobiology (Benner et al., 2004; Boska et al., 2005; Reynolds et al., 2007b; Benner et al., 2008; Brochard et al., 2009; Niwa et al., 2012; Saunders et al., 2012). Each is capable of affecting the brain microenvironment and, in specific instances of therapeutic immune modulation, can improve the overall disease course (Reynolds et al., 2007a; Reynolds et al., 2007b; Reynolds et al., 2010; Mosley and Gendelman, 2017). Thus, with broad recent advancements in the understanding of neuroimmunity and the linkages to neuroimmune pharmacology, the prospect to harness peripheral immunity in slowing or even halting neurodegenerative activities are now within reach.

This possibility extends far beyond PD. The need to develop treatments for other neurodegenerative diseases, such as Alzheimer’s disease (AD), stroke, and traumatic brain injury (TBI), cannot be understated. As in PD, increased incidence observed due to our aging population can also be seen in AD. Between the years 2000 and 2014, AD-related death increased by 89% (Weller and Budson, 2018). Additionally, the annual cost of AD has grown to nearly $500 billion (Takizawa et al., 2015). The cost of TBI is around $60 billion annually, affecting approximately 1.7 million people in the US alone and representing 30% of injury-related deaths (Langlois et al., 2006; Roozenbeek et al., 2013; Nguyen et al., 2016). While stroke is a common leading cause of death, survivors will often experience long-term disability and cognitive impairment (Kalaria et al., 2016). Despite these statistics showing increased prevalence of neurological disease and injury, therapeutics for are mainly palliative and none successfully address the chronic neuroinflammation that allows neurodegenerative disease to progress.

It is our intent for this review to summarize the relationships among PD pathobiology, immunology, and neurodegeneration with a particular focus on the role of adaptive immunity in disease pathology and therapy. These relationships will then be further explored in other disease states that possess a similar neuroinflammatory pathology. In addition, we will discuss the current symptomatic therapies routinely administered to patients with PD and other neurodegenerative disorders to emphasize the need for curative interventions capable of targeting pervasive, chronic neuroinflammation. We posit that aggregated and misfolded proteins break immune tolerance, which leads to immune system activation and cellular imbalance perpetuating neurodegeneration. Our focus is therefore on the development of immunomodulatory agents to treat progressive neurodegenerative disorders such as PD and expand such therapy into other neurodegenerative diseases that share a similar neuroinflammatory phenotype.

## 2. Pathology of Parkinson’s disease

**Fig. 1.**
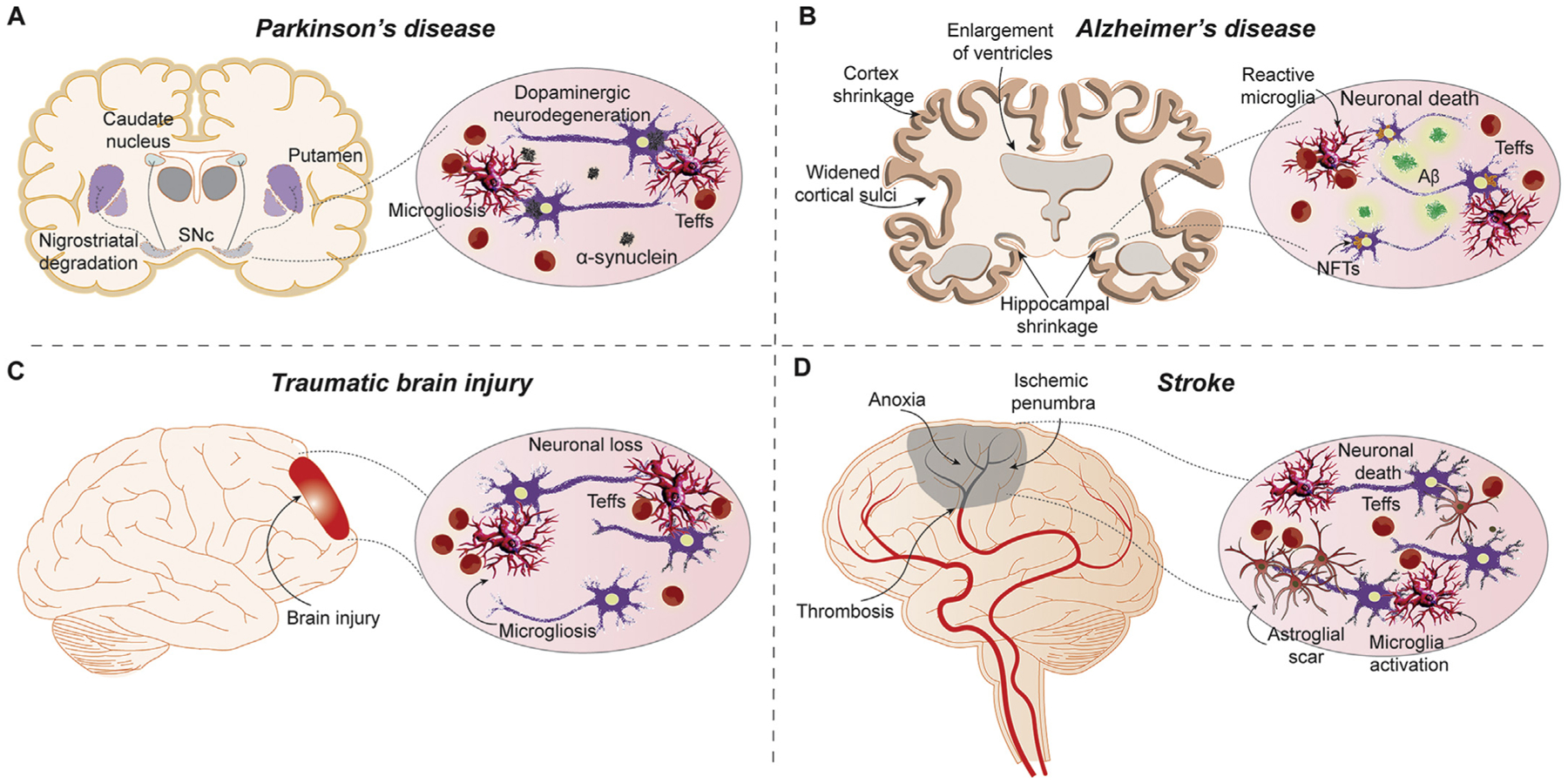
Immunity and neurodegenerative disease pathogenesis. Neurodegenerative processes often involve interactions between infiltrating effector T cells (Teffs) and microglia to affect the progression of neurodegenerative disease either due to aberrant protein processing or danger/damage signaling due to neuronal injury and death. Microglia respond to a reactive phenotype during cell-cell interactions with Teffs after migrating across the blood brain barrier (BBB). While the pathological consequences and origins of such neuroinflammation varies across disease states, the inflammatory signature shared by the represented neurodegenerative diseases identifies a common target for disease therapy. (A) In Parkinson’s disease, α-syn is modified, misfolded, oligomerized, and released into the extracellular environment following neuronal injury. Misfolded and oxidatively modified α-syn aggregates and elicits microglial activation that perpetuates neuronal damage within the substantia nigra pars compacta (SNc). Modified self-proteins, such as aggregated N-α-syn with proinflammatory milieu, initiate a systemic, adaptive immune response following their drainage to secondary lymphoid tissues. Increased BBB permeability and Teff influx exacerbate neuroinflammation and neurodegeneration upon recognition in the CNS through the secretion of inflammatory mediators that shift the brain microenvironment towards a pro-inflammatory phenotype. (B) In Alzheimer’s disease, inflammation-associated neuronal death results in the release of amyloid-β (Aβ) plaques and neurofibrillary tangles (NFTs) into the extracellular environment which induce inflammation and subsequent neural death. Increased inflammation augments aberrant amyloid protein processing, Aβ accumulation, and hyperphosphorylation of Tau. (C) In TBI, trauma to the brain causes tissue damage, which in turn activates microglia and induces Teff influx. Microglia shift from a homeostatic to a reactive phenotype with secretion of proinflammatory factors leading to increased overall cytotoxicity that propagates neuroinflammation and neurodegeneration. (D) Following an ischemic stroke, tissue damage and BBB injury initiate microglial activation and influx of pro-inflammatory immune cells. Teffs infiltrating the brain, secrete neurotoxic and inflammatory mediators alongside reactive microglia resulting in penumbric spreading of neuronal death and astroglial scarring.

While the principal pathology of PD involves the loss of nigrostriatal dopaminergic neurons and their efferent termini, another pathological hallmark of PD is the misfolding, oligomerization, and accumulation of alpha-synuclein (α-syn) and other proteins within nigrostriatal neurons (Fig. 1A) (Goedert et al., 2013). Under homoeostatic conditions, misfolded proteins are ubiquitinated, directed to the proteasomes, and degraded for elimination or amino acid recycling. However, ubiquitin-proteasome processes fail during PD and allow α-syn misfolding, ubiquitination, and oligomerization without elimination. Subsequent accumulation of misfolded α-syn oligomers eventually leads to the formation of inclusions or Lewy bodies within affected dopaminergic neurons, which is an intracellular response thought to sequester proteins that cannot be otherwise eliminated (Holdorff, 2002; Volpicelli-Daley et al., 2011; Dryanovski et al., 2013). However, the presence of misfolded and modified α-syn, caused by unknown disease processes, underlies the neurological impairments associated with disease processes (Outeiro et al., 2019). While Lewy bodies themselves are considered to be relatively non-toxic, oligomerization of α-syn species forms toxic fibril structures that eventually are inserted into neuronal plasma membranes leading to lipid bilayer penetration, pore formation, and neuronal injury due to perturbation of homeostatic calcium ion influx and oxidative stress (Conway et al., 1998; Conway et al., 2000; Choi et al., 2012; Nakamura, 2013; Fusco et al., 2017). With increasing oxidative stress, post-translational modifications of α-syn become more prevalent leading to increased levels of misfolding and accumulation of α-syn and toxic fibril forms (Hashimoto et al., 1999; Souza et al., 2000; Scudamore and Ciossek, 2018). As membrane penetration of toxic fibrils increase with subsequent neuronal injury, membrane leakage and neuronal lysis follow with release of the misfolded, modified, and oligomerized α-syn into the brain parenchyma. Modified and misfolded α-syn species function as danger/damage-associated molecular patterns (DAMPs) that activate myeloid phagocytes (e.g., microglia, macrophages, and dendritic cells) via pattern recognition receptors (PRRs) such as CD36, toll-like receptor 2 (TLR2), TLR4/CD14, and CD11b (Croisier et al., 2005; Reynolds et al., 2007a; Zhang et al., 2007; Benner et al., 2008; McGeer and McGeer, 2008; Su et al., 2008; Fellner et al., 2013; Kim et al., 2013; Hou et al., 2018). Thus, PRR recognition of damage-associated signals may play a pivotal role in the establishment and perpetuation of oxidative stress and inflammation under the parkinsonian condition.

Additional factors affecting the vitality of dopaminergic neurons are the excessive production of reactive oxygen and nitrogen species (ROS and RNS) (Stone et al., 2009). In a healthy nervous system, a balance is maintained between reactive species production and clearance; however, in PD, an imbalance ensues whereby ROS/RNS production outweighs its clearance. The unpaired electrons in ROS make them highly reactive to the surrounding molecular milieu. For instance, as dopamine is a relatively unstable molecule and undergoes auto-oxidation, it is susceptible to ROS-mediated substitution with the formation of dopamine quinones that can form dopamine adducts with proteins and other biomolecules that affect structural proteins or enzyme functions (Hastings, 2009; Koppula et al., 2012). Notably, with increased oxidative stress and inflammation, excess production of oxygen radicals and nitric oxide reactants increases the intracellular formation of reactive peroxynitrite in dopaminergic neurons (Floor and Wetzel, 1998; Reiter et al., 2000; Ebadi and Sharma, 2003). Through reactions with peroxynitrite, tyrosine and cysteine moieties are readily nitrated or nitrosylated, respectively, on proteins, such as α-syn, parkin, DJ-1, and Pink1, leading to misfolding, loss of function, and neuronal injury with subsequent neuronal death (Viner et al., 1999; Reiter et al., 2000; Danielson and Andersen, 2008). Thus, ROS/RNS and peroxynitrite formation represent an integral component of oxidative stress and neurodegeneration in several models of PD (Gupta et al., 2014). For instance, in the 1-methyl-4-phenyl-1,2,3,6-tetrahydropyridine (MPTP) animal model of PD, 1-methyl-4-phenylpyridinium (MPP+) neurotoxin accumulates in the mitochondria of dopaminergic neurons where it binds and reduces the activity of complex I (Langston, 2017). Reduction of complex I activity decreases ATP synthesis and generates ROS, which is thought to be a first-hit in the neurodegenerative process (Koppula et al., 2012; Langston, 2017). Additionally, MPP+ causes dopamine to be expelled from the intracellular vesicles to the cytoplasm, allowing auto-oxidation of dopamine into toxic ROS with formation of dopamine-quinone, hydrogen peroxide, and superoxide radicals. These enable its metabolism into hydrogen peroxide by monoamine oxidase (MAO) (Lotharius and Brundin, 2002; Langston, 2017).

ROS/RNS also act as major regulators of neuroinflammation with excessive amounts leading to a proinflammatory and neurodestructive environment within the brain. Moreover, the SNc is particularly susceptible to ROS/RNS damage due, in part, to sparse antioxidant defenses in that area of the brain and decreased levels of glutathione (GSH), a necessary ROS/RNS scavenging agent (Smeyne and Smeyne, 2013). Interestingly, RNA signatures such as leucine-rich repeat kinase 2 (*LRRK2*) G2019S mutation predisposes to PD as do microRNA (miRNA) and piwi-interacting RNA (piRNA) alterations as observed in PD and AD patients (Qiu et al., 2014; Roy et al., 2017). Specifically, in midbrain neurons of PD patients, the pathway regulating peroxisome proliferator-activated receptor-gamma coactivator (PGC-1α), a transcriptional coactivator involved in mitochondrial processes, stress-induced apoptosis, and cAMP response element-binding protein (CREB) function, is inactivated, indicating mitochondrial dysfunction and another potential source of oxidative stress in PD (Liu and Lin, 2011; Schulze et al., 2018). In total, the initial neuronal damage and injury with ensuing release of misfolded and modified proteins play a principal role in the elicitation of proinflammatory responses mediated primarily by microglia in PD, which will be further explored in later sections (Tansey and Goldberg, 2010; Gelders et al., 2018).

## 3. Neurodegenerative disease pathology

AD is characterized and staged by levels of dementia and represents 80% of patients diagnosed with dementia (Crous-Bou et al., 2017). Similar to PD, AD is driven by a combination of both genetic and environmental factors (Lane et al., 2018). The most prominent pathological features of AD are neurofibrillary tangles (NFTs), amyloid plaques (Aβ), neuronal injury, and neurodegeneration (Fig. 1B) (Jack Jr. et al., 2018). Fibrillogenic species of Aβ are deposited in the neuron and membrane leading to Ca^2+^ influx and excitotoxicity, while hyperphosphorylation of tau protein leads to loss of microtubule binding capability with loss of microtubule stability and support, both combining to promote cell death (Pooler et al., 2013). The neurons then release their contents into the brain’s microenvironment. Similar to modified and misfolded α-syn, NFTs and Aβ proteins are recognized as foreign by PRRs that induce an immune response and activate microglia. This leads to the production of pro-inflammatory neurotoxins, excitotoxicity, and oxidative stress; all contributing to continued neurodegeneration (Gendelman and Mosley, 2015).

The pathology of TBI is complicated by the many permutations of multiple primary lesion types, such as intracranial hemorrhages, contusions, hematomas, and direct axonal damage (Maas et al., 2008) as well as secondary injuries, all of which can develop from minutes to months after the primary injury (Jassam et al., 2017). During and after formation of the primary lesion, pro-inflammatory signals are immediately released, causing microglia to become activated and proliferate as well as activate astrocytes which also secrete pro-inflammatory cytokines (Fig. 1C). Additionally, cellular debris, pro-inflammatory mediators, and DAMPs that are released within the brain parenchyma migrate to the peripheral circulation and secondary lymphoid tissues and elicit systemic inflammatory responses (Jassam et al., 2017). Secondary injuries, mediated by excitotoxicity, free radical generation, and local and systemic pro-inflammatory activated immune cells, traffic back to encephalitogenic foci to permeate the parenchyma, perpetuate neuroinflammation, and subsequently lead to neurological impairment (Faden et al., 1989; Anthonymuthu et al., 2016; Dorsett et al., 2017; Simon et al., 2017).

Strokes broadly consist of decreased blood supply to a specific brain region, where neurons are lost at the rate of 1.8 million per minute without treatment (Fig. 1D) (El-Koussy et al., 2014). This decrease in blood supply is due to arterial thrombosis, which blocks blood flow to the affected region known as an infarct, wherein damage is typically irreversible and anoxia is pervasive. The neurons surrounding the infarct make up the ischemic penumbra, otherwise known as tissue-at-risk (El-Koussy et al., 2014). The infarct can expand into this region given enough time, increasing the area of irreversible damage. Moreover, if a large enough vessel is blocked, patients are at risk for severe neurological deficits and poor prognosis without early treatment.

## 4. Peripheral immunity and brain homeostasis

**Fig. 2.**
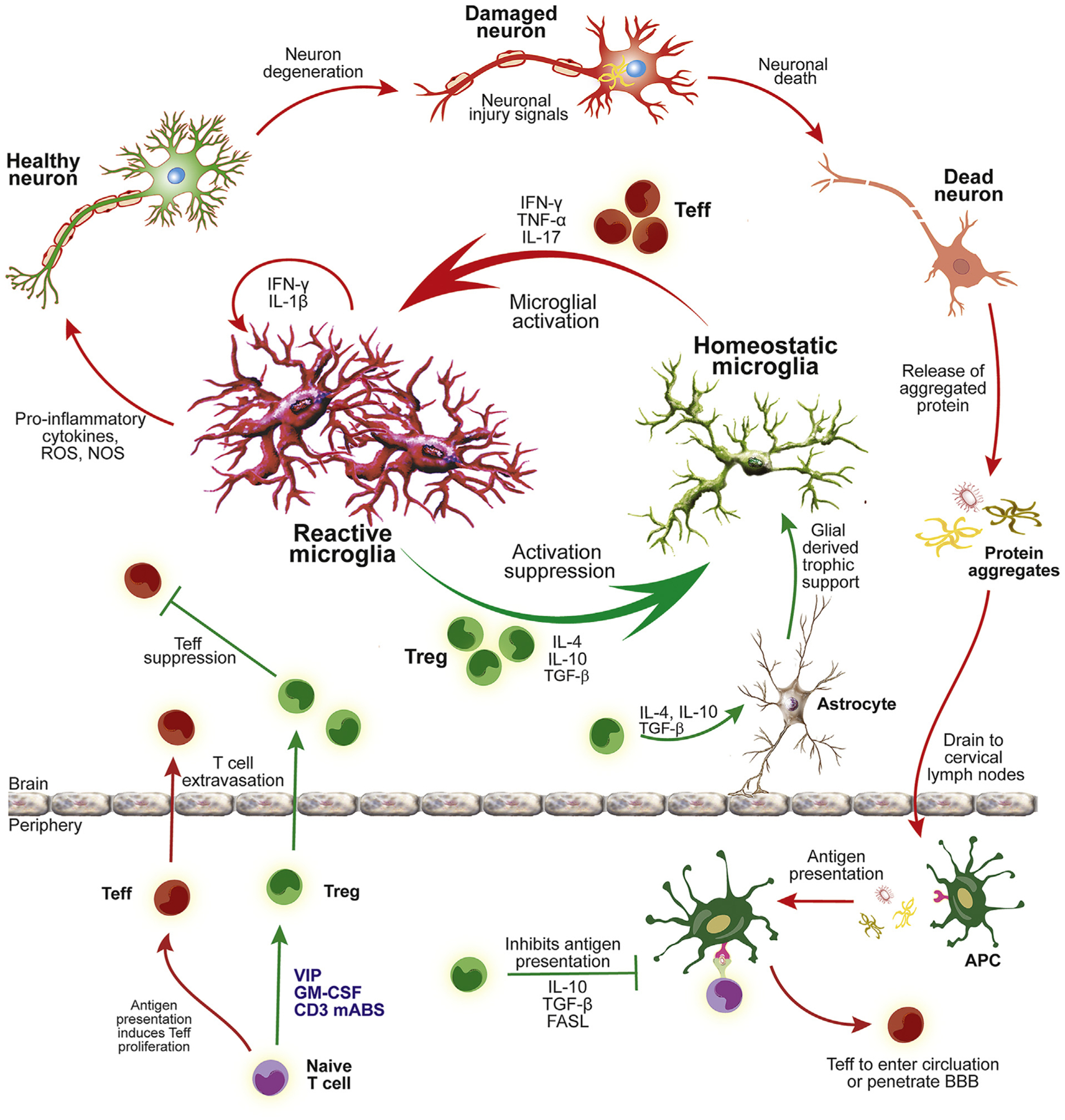
Therapeutic transformation of the brain’s microenvironment in neurodegenerative disease. A neuroinflammatory microenvironment heralds the onset and progression of neurodegenerative disease. Inflammation perpetuates microglial activation and subsequent neurodegeneration and is pivotal in the progression of Parkinson’s disease, Alzheimer’s disease, stroke, and traumatic brain injury. In most neurodegenerative disorders, aberrant protein modifications and misfolding allow fibrillogenic and aggregated forms to be released into the extracellular environment. Similarly in TBI and stroke, damage/danger signals from tissues and neurons are also produced and released upon injury. Modified proteins and DAMPs initiate the activation of microglia with the production and release of inflammatory mediators and cytokines as well as reactive oxygen and nitrogen species. These prove to be neurotoxic with the ability to damage surrounding neurons. Additionally, the proinflammatory milieu drains to secondary lymphoid tissues where it activates antigen presenting cells (APCs) to present modified self-antigens such as nitrated α-synuclein. Under the influence of proinflammatory co-stimulatory signals, naïve T cells initiate programs to differentiate and expand into pro-inflammatory effector T cells (Teffs) such as Th1 and Th17 cells. These Teffs extravasate across the blood brain barrier at inflammatory foci whereby Teffs are reactivated by microglia or macrophages which exacerbate neuroinflammation and neurodegeneration through the secretion of inflammatory mediators. Overall, Teff-microglia interactions shift the brain microenvironment towards a pro-inflammatory neurotoxic environment that hastens disease progression. On the other hand, regulatory T cells (Tregs) have the capacity to harness microglial and APC activation, attenuate inflammation, inhibit Teff induction, and induce astrocytic neurotrophins, thus effectively transforming a neurotoxic environment to a neurotrophic state. In chronic neurodegenerative disorders and acute CNS damage, Treg processes are often overwhelmed due to their low numbers or dysfunction. Therefore immune modulating agents, such as anti-CD3 mAbs, vasoactive intestinal peptide (VIP), or granulocyte macrophage colony stimulating factor (GM-CSF) are utilized to increase Treg number and function which also can extravasate at sites of neuroinflammation and execute Treg processes to rebalance the neurotoxic state to one of neuroprotection.

Immune responses generated in either the CNS or periphery have historically been perceived as relatively distinct processes by virtue of anatomical barriers. However, immunological communication between the two compartments is allowed to the extent that CNS pathogens are encountered and eliminated, thus negating the concept of immunological privilege within the CNS. This is underscored in neuroinflammatory disease states, which implicate dysregulated communication accompanied by systemic immune responses from which activated immune cells can migrate to areas of inflammation in the brain (Fig. 2). We posit that the pathogenesis of PD involves cyclic phases of neurodegeneration and neuroinflammation, where the proteasome-ubiquitin system fails to clear excess α-syn. Subsequently, α-syn misfolds and accumulates in neurons in the SNc resulting in neurodegeneration. Neuronal degeneration leads to release of α-syn which is oxidatively modified, misfolded, and oligomerized, and triggers the activation of microglia (Lim and Tan, 2007; Thomas et al., 2007). This innate response shifts the microenvironment of the brain into a proinflammatory state, creating a cycle of neuroinflammation, protein misfolding, and neurodegeneration. As microglia are heavily concentrated in the SNc relative to other parts of the brain, the chronic activation of these CNS-resident immune cells has been implicated in the specific degeneration of localized dopaminergic neurons (Lawson et al., 1990; Kim et al., 2000; Bachiller et al., 2018). While activated microglia secrete a variety of pro-inflammatory factors, such as tumor necrosis factor-α (TNF-α), interleukin-1β (IL-1β), IL-6, IL-12, nitric oxide (NO), prostaglandin E2 (PGE2), and superoxide radicals, they are also sensitive to gut-derived lipopolysaccharide (LPS), interferon-γ (IFN-γ), β-amyloid, CD40L, gangliosides, and various chemokines and neurotransmitters (Suzuki et al., 2005; Stone et al., 2009). Activation of microglia resulting from interactions with these substances can increase secretion of pro-inflammatory factors leading to upregulation of complement receptors and cell adhesion molecules that exacerbate dopaminergic-associated neurotoxicity (Kreutzberg, 1996).

The resultant inflammation associated with microglial activation increases the permeability of the blood-brain barrier (BBB) and enables extravasation of adaptive immune cells due, in part to chemoattractant gradients generated from inflammatory foci within the brain (Garretti et al., 2019). This increase in BBB permeability, as well as T and B cell infiltration, has been noted in animal models of PD, such as 6-OHDA, MPTP, and AAV2-Syn models (Carvey et al., 2005; Theodore et al., 2008; Brochard et al., 2009; Reynolds et al., 2010; Zhang et al., 2017). These adaptive immune cells are located in higher numbers in the SN of people with PD compared to healthy controls, specifically near neuromelanin-containing dopaminergic neurons (McGeer et al., 1988; Brochard et al., 2009; Garretti et al., 2019). Additionally, Sulzer et al. (2017) reported that PD patients’ peripheral blood T cells recognized α-syn, with most secreting predominantly IL-5 and a small population secreting IFN-γ. The location of immune cells within the SNc and T cell recognition of α-syn-derived epitopes suggest that adaptive immune cells may target dopaminergic cell-derived epitopes, thus implicating an autoimmune response mounted against α-syn (Mosley and Gendelman, 2017; Sulzer et al., 2017; Garretti et al., 2019). This immune cell infiltration propagates inflammation in other regions of the CNS and perpetuates a positive feedback loop of innate immune cell activation, protein misfolding, and neuronal death throughout the CNS (Kannarkat et al., 2013). Interestingly, this cycle is demonstrated in the α-syn overexpression mouse model where increased BBB permeability, adaptive immune cell infiltration, and microglial activation appear before neurodegeneration occurs, suggesting these play causal, rather than consequential roles in neurodegeneration (Theodore et al., 2008; Garretti et al., 2019).

Post-translationally modified neuronal proteins from dying dopaminergic cells also influence disease progression. The pro-inflammatory influence of nitrated α-syn (N-α-syn) is not restricted to innate immune activation or localized to the brain. Failure of ubiquitin-proteasome system to clear N-α-syn extends the formed aggregates beyond the CNS (Lim and Tan, 2007; Thomas et al., 2007). These aberrant forms of N-α-syn oligomers also traverse throughout the body as well. Antigen presentation of endocytosed modified self-protein components in the context of major histocompatibility complex class II (MHC-II) molecules by CNS resident microglia and/or infiltrating innate immune cells can act as a critical determinant of an adaptive immune response, initiating T cell-mediated neurotoxicity. Notably, the dopaminergic neurons of MHC-II^−/−^ mice in the AAV2-SYN overexpression model were spared, suggesting a necessary role of MHC-II in neurodegenerative diseases (Harms et al., 2013; Jimenez-Ferrer and Swanberg, 2018; Lindestam Arlehamn et al., 2019). Additionally, genome-wide association studies (GWAS) have associated sporadic PD with two HLA class II alleles, DRB1*15:01 and DRB5*01:01. For example, an increased expression of DRB1*15:01 could lead to an increase in HLAs with an increased specificity for α-syn or a general pro-inflammatory state in people with PD (Garretti et al., 2019).

Adaptive immune responses that are associated with the brains of PD patients are not restricted to the CNS and most likely are initiated in the periphery. For instance, N-α-syn and the proinflammatory milieu from the brain drain to cervical lymph nodes, where it initiates a peripheral, adaptive immune response (Benner et al., 2008; Stone et al., 2009). Antigen-presenting cells (APCs), such as dendritic cells and macrophages, activated by DAMPs and proinflammatory mediators, process N-α-syn and present the modified self-epitopes in the context of MHC-II molecules. Naïve T cells, via T cell receptors that recognize the modified self-epitope-MHC-II complex on APCs, initiate programs of differentiation that along with appropriate costimulatory molecules and secondary signals, differentiate into effector T cells (Teffs), which expand into programmed T cell subtypes characteristic of pro-inflammatory Teffs, such as Th1 and Th17 cells (Kannarkat et al., 2013). Peripherally induced Teffs can then permeate the BBB and provoke inflammation along the nigrostriatal axis by recognition of their cognate modified self-antigens/MHC-II complex. When modified self-antigens such as N-α-syn are then presented by MHC on activated microglia or macrophages in the CNS, T cells are reactivated and expanded, and pro-inflammatory Teffs express a neurotoxic program. Th1-sourced IL-2, IFN-γ, and TNF-α exacerbate neurotoxicity by inducing microglia to release ROS and NO, while the production of IL-17A, IL-17F, IL-21, IL-22, and TNF-α by Th17 cells further the inflammatory environment in the CNS (Kosloski et al., 2010; Tansey and Goldberg, 2010). While both Th1 and Th17 Teffs have been shown to exacerbate neuroinflammation and dopaminergic neurodegeneration, Th17 effectors yield significantly greater neurotoxic potential than Th1 Teffs (Reynolds et al., 2010). Additionally, Th17 cells have been shown to directly cause damage to dopaminergic neurons derived from human iPSC-derived midbrain neurons (Sommer et al., 2018). Thus, this persistent inflammatory cycle perpetuates neurodegeneration and microglial activation towards a phenotype indicative of chronic inflammation.

The dynamics in immune responses between pro-inflammatory neurotoxic activities and regulatory neurotrophic CD4+ T cells determine disease progression. Over expression of neurotoxic activities and failures of neurotrophic molecules to regulate the cycle perpetuates inflammation-induced neurodegeneration and has recently been uncovered as a hallmark of human disease. Specifically, regulatory T cells (Tregs) capable of abrogating neuroinflammation and resultant dopaminergic neuron loss are functionally stunted in PD patients exhibiting decreased capability to suppress CD3/CD28-induced Teff proliferation (Saunders et al., 2012). Moreover, Teff numbers were increased in PD patients compared to controls and directly correlated with the severity of motor function, while Treg dysfunction was also linked with decreased motor function and increased disease severity. These data suggested that increased Teff function and/or corresponding decrease in Teff regulation by Tregs, accelerated or intensified disease progression as determined by clinical motor function scores. Understanding the mechanisms by which Tregs exercise suppressive effects is continually evolving. Tregs have been shown to suppress specific immune responses by induction and release of anti-inflammatory cytokines such as IL-10 and TGF-β, inhibition of antigen presentation, disruption of Teff induction and metabolism by suppressing antigen presentation, removing IL-2 from Teffs, Fas-FasL-mediated killing of pro-inflammatory Teffs and reactive microglia (Fig. 2) (Benner et al., 2004; Reynolds et al., 2007b; Reynolds et al., 2009; Stone et al., 2009). Moreover in the CNS, Tregs seemingly have the ability to induce astrocytes to produce neurotrophic factors. As intuitively hypothesized, adoptive transfer of Tregs elicits neuroprotection in animal models of PD, while transfer of Teffs exacerbates neurodegeneration (Benner et al., 2004; Reynolds et al., 2007b; Benner et al., 2008; Brochard et al., 2009; Reynolds et al., 2010). Across studies, elevated levels of pro-inflammatory cytokines such as IL-2, IL-6, IL-8, TNF-α, and IFN-γ have been reported in the blood of PD patients, with cytokine concentrations correlating with clinical stage and disease progression (Reale et al., 2009; Garretti et al., 2019). Together, these data indicate an imbalance in innate and/or T cell populations and functions among PD patients relative to healthy controls, and are congruent with increases in inflammatory helper T cell populations, specifically Th1 and Th17, within an environment of fewer naïve T cells and reduced anti-inflammatory capacities (Bas et al., 2001; Chen et al., 2015).

The observation of chronic inflammation in neurodegeneration is not exclusive to PD. In Alzheimer’s disease (AD), RORγ- or IL-17-expressing Th17 Teffs were significantly increased in AD patients with mild cognitive impairment (MCI) compared to control populations (Saresella et al., 2011; Oberstein et al., 2018). On the other hand, no significant differences in Treg populations were discerned among any AD group compared to controls, however peripheral blood Treg frequencies in AD patients positively correlated with CSF levels of total Tau, phosphorylated Tau, and Aβ40. Another study concluded that frequencies of Tregs and resting Tregs (CD45RA+/CD25dim) were diminished, while no differences were detected among activated (CD45RA−/CD25bright) and secreting (CD45RA-/CD25dim) Tregs (Ciccocioppo et al., 2019). However, none of these studies assessed the suppressive capacity of Tregs in AD. Together, these data suggest that in AD, altered Treg phenotypes and possibly function could contribute to neuroinflammation and disease progression. In ischemic stroke and TBI, adaptive immune responses also play a role in the pathophysiology. Injured brain tissues initiate an inflammatory cascade that upregulates Teffs and pro-inflammatory cytokines, both temporally and spatially proximal to injury, and eventually lead to microglial activation and neuroinflammation (Kelso and Gendelman, 2014; Picascia et al., 2015). Pathobiological commonalities associated with such degenerative and traumatic diseases of the CNS pave the way for therapeutic approaches that target inflammation by means of Treg induction. As the immune components in several of these neurodegenerative disorders become increasingly better defined, investigations seeking to elucidate broadly applicable immune-modulating therapies that target restoring regulatory capacity and restraining associated neurodegenerative processes are being explored.

## 5. Current clinical approaches

Current clinical interventions that are curative for neurodegenerative diseases are lacking as they fail to address causative aspects of the diseases. Most therapies are for the most part palliative in nature. For AD, the available therapies aim to enhance the quality of life of the patient, but do not alter disease progression or slow the rate of decline (Weller and Budson, 2018). One of the most prominent therapy includes is cholinesterase inhibitors, such as donepezil, rivastigmine, and galantamine and are indicated for mild, moderate, and severe AD, respectively (Howard et al., 2012). Memantine is indicated for patients with moderate to severe AD dementia for patients who experience attention and alertness difficulties (Grossberg et al., 2013) and acts as a non-competitive N-methyl-D-aspartate receptor antagonist as well as a D2 receptor agonist. Several therapeutic strategies target the production or clearance of Aβ plaques and NFTs, but these have not shown promising results and many have been demonstrated to be ineffective (Doody et al., 2014; Salloway et al., 2014; Weller and Budson, 2018).

Stroke and TBI have even fewer options available. The gold standard for stroke treatment is tissue plasminogen activator (tPA), which is severely limited by a narrow therapeutic time window and adverse side effects (Knecht et al., 2017). Several pharmacological treatments attempted to address these two drawbacks of tPA, but medications aimed at restoring neurological functions have shown limited efficacy. Several medical interventions to address the anatomical damage of TBI have been attempted, but currently no effective treatments tackling the neuronal damage and chronic inflammation are available (Galgano et al., 2017). Ongoing experimental studies for pharmaceutical neuroprotective treatments have not yet been translated into clinical therapies, and those in clinical trials have not shown promising results. Due to the lack of availability and limited efficacy of current approaches, shifting focus to target the immune microenvironment of the brain could prove beneficial.

Currently, the majority of treatments available for patients with PD are entirely palliative and designed to either increase or replace dopamine. However, none of these symptomatic strategies slow or halt disease progression. The most widely used and effective of these medications is levodopa, a dopamine precursor that crosses the BBB and is converted to dopamine in the brain. While novel formulations have enabled optimal dose release, better brain-targeting delivery, and a reduction in side effects such as dyskinesia and nausea, continual treatment can reduce the stability of levodopa’s benefit (Dong et al., 2016). Even when paired with catechol O-methyltransferase (COMT) inhibitors to prolong levodopa in circulation, off-time is only just decreased, while both motor and non-motor symptoms inevitably return (Schrag, 2005; Ferreira et al., 2010).

Monoamine oxidase B (MAO-B) inhibitors have also proven efficacious in reducing symptoms associated with PD as an early stage monotherapy or in conjunction with levodopa. MAO-B inhibitors protect endogenous dopamine by preventing degradation by MAO-B, which metabolizes dopamine in the presynaptic neuron. Unfortunately, dangerous xenobiotic interactions are a potential issue if patients consume tyramine-rich foods or ingest drugs containing sympathomimetic or serotoninergic substances (Riederer and Laux, 2011). Inhibition of MAO-B in tyramine or serotonin metabolism would increase tyramine and serotonin levels leading to hypertenisive crisis or serotonin syndrome. Dopamine receptor agonists that target upregulated and sensitized post-synaptic receptors are indicated to improve motor fluctuations for PD patients. However, excessive receptor activation can lead to adverse symptomatic side effects such as hallucinations, low blood pressure, gambling addiction, compulsive spending, and hypersexuality (Moore et al., 2014). To correct the imbalance between dopamine and acetylcholine in the pathology of PD, muscarinic acetylcholine receptor antagonists have been included in symptomatic treatment regimens. Clinical use of anticholinergics, however, is sparse in PD. The potential for adverse events is currently high as anticholinergic application has been found to increase gait freezing, confusion, hallucinations, and the rate of falls in more elderly patients (Wawruch et al., 2012).

Recent advancements in scientific understanding and technological innovation have opened doors to new treatment strategies that are not solely symptomatic. As opposed to increasing dopamine and dopamine-associated signaling, current treatment strategies seek to regenerate dopaminergic neurons that are selectively lost in PD or elicit neuroprotection via immunotherapy to prevent dopaminergic neuronal death. While stem cell-based therapies for PD are nearing use in clinical trials, cell replacement therapy focused on rescuing striatal dopaminergic deficits raises concerns regarding variable clinical benefits and tolerability within a heterogeneous cohort of PD patients (Henchcliffe and Parmar, 2018). The surgical nature of the treatment and the current expense associated with such intensive therapy also complicate the current application of pluripotent stem cells in PD. Moreover, neuronal replacement therapy by stem cell transplantation does not entirely alleviate parkinsonian pathology. In PD patients transplanted with fetal stem cells, aberrant α-syn species, Lewy body pathology, and reactive microglia were associated with the donor stem cell-derived dopaminergic neurons obtained at post-mortem examination of the recipients years after transplantation (Kordower et al., 2008; Li et al., 2008; Mendez et al., 2008). Notably, further examination revealed that transplants with Lewy pathology and aggregated α-syn were associated with reactive microglia. This suggested that non-autonomous cell processes, including innate and/or adaptive cell-mediated immunity, contribute to neurodegenerative processes in PD (Dawson, 2008).

While the inflammatory brain microenvironment in PD exacerbates oxidative stress, protein misfolding, neurotoxicity, and neurodegeneration, Treg function has been shown to attenuate this neuroinflammatory cascade (Benner et al., 2004; Reynolds et al., 2007b; Reynolds et al., 2010; Kleinewietfeld and Hafler, 2014; Mosley and Gendelman, 2017; Keeler et al., 2018). As discovery of translatable Treg inducing agents expands, so does the list for testable therapies for neuroinflammatory diseases. Other Treg inducers that are currently being investigated for their therapeutic potential in PD are histone deacetylase (HDAC) inhibitors, glucocorticoids (GCs), anti-CD3 monoclonal antibodies (mAbs), retinoic acid (RA), granulocyte macrophage-colony stimulating factor (GM-CSF), rapamycin, and neuropeptides such as vasoactive intestinal peptide (VIP) (Benner et al., 2004; Battaglia et al., 2006; Reynolds et al., 2007b; Wang et al., 2009; Liao et al., 2010; Reynolds et al., 2010; Bach, 2011; Hall et al., 2011; Souza-Moreira et al., 2011; Zou et al., 2011; Kosloski et al., 2013; Olson et al., 2015; Gendelman et al., 2017). As Treg induction and adoptive transfer have been assessed for safety, feasibility, and therapeutic efficacy in solid organ transplantation, graft versus host disease (GvHD), autoimmunity, ALS, and PD (Desreumaux et al., 2012; Gendelman et al., 2017; Romano et al., 2017; Vaikunthanathan et al., 2017; Thonhoff et al., 2018), additional clinical investigations are necessary to further explore Treg-mediated modalities as efficacious immunotherapeutic strategies in PD and other neurodegenerative diseases that possess similar neuroinflammatory signatures, such as AD, TBI, and stroke

## 6. Immunotherapy

Immunotherapy is most commonly used in cancer treatment where an overall survival benefit has been demonstrated (Van Limbergen et al., 2017). Immunotherapeutic strategies in cancer enhance the immune system to mount a response against the tumor, while minimizing immune suppression (Banerjee et al., 2018). In PD and other neuroinflammatory-based neurodegenerative disorders, the immune system is in an activated state that generally exacerbates neurotoxicity and neurodegeneration, thus immune suppression would confer potential therapeutic benefits. In the reverse manner, whereby immunotherapy is used to exacerbate the immune response, it can also quell the activated immune state in the CNS. Thus, the goal for immunotherapeutic intervention is to attenuate neuroinflammation and induce a neuroprotective environment within the CNS that prevents further neurodegeneration and diminishes disease progression and clinical symptoms. Whether neuroimmunotherapy contributes directly to processes that induce endogenous neuronal regeneration is contentious (Farzanehfar, 2018), however, such interventions will play a significant role in homeostatic maintenance and create a neuroprotective environment that is beneficial for neuronal replacement or regenerative therapy. One strategy to accomplish this is manipulating the patient’s immune system, subsequently altering the CNS microenvironment to provide greater anti-inflammatory and neuroprotective conditions. The pharmaceutical induction of Tregs with increased function impede the perpetual cycle of neurodegeneration and neuroinflammation (Fig. 2). This has been shown to provide neuroprotection to surviving dopaminergic neurons and improve clinical scores and outcomes beneficial to PD patients (Reynolds et al., 2007b; Reynolds et al., 2010; Mosley et al., 2012; Gendelman et al., 2017; Mosley and Gendelman, 2017). Such drugs are currently being studied in a variety of PD associated contexts.

**Fig. 3.**
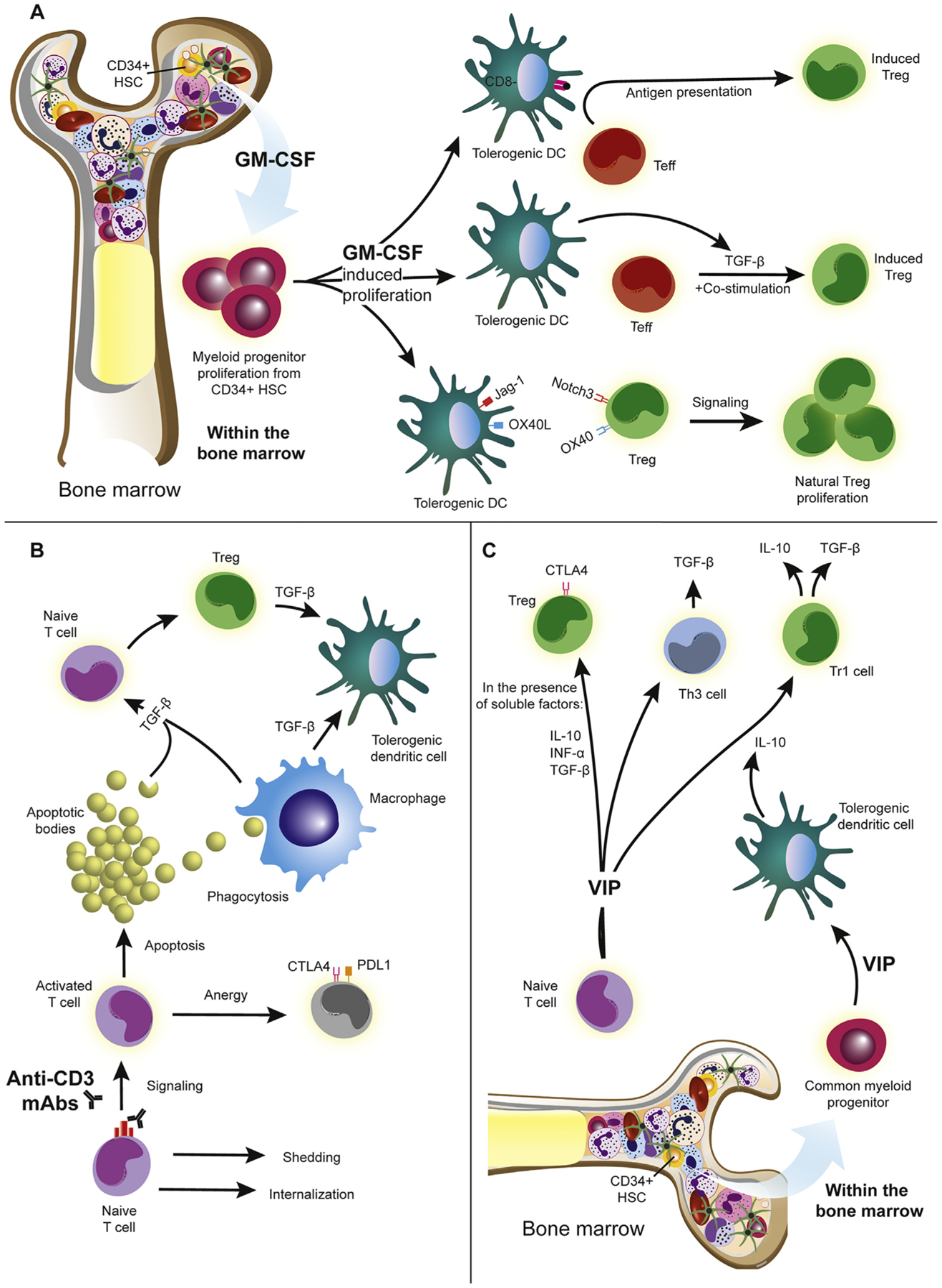
Mechanisms underlying Treg expansion. The growing body of research that implicates immunological dysfunction in the pathology of Parkinson’s disease and other neurodegenerative disorders has shed light on new pharmacological targets capable of addressing more causal aspects of the neurodegenerative disease. Specifically, Treg inducing agents capable of shifting the immune environment towards an anti-inflammatory, neuroprotective state are being explored for future clinical application. (A) GM-CSF acts as a Treg inducing agent by initially facilitating the differentiation of bone marrow progenitor cells into tolerogenic dendritic cells (DCs). These dendritic cells express the surface markers OX40L and Jagged-1 (Jag-1) which induce and expand natural Treg and induce Treg proliferation following interactions with their cognate receptors. TGF-β secreted by the BMDCs enable the conversion of Teffs into Tregs in the presence of co-stimulatory molecules. A specific population of tolerogenic DCs that are CD8α- are remarkably responsive to GM-CSF as they expand in both number and function and directly transform Teffs to Tregs. (B) In contrast to other immunomodulating therapies, anti-CD3 monoclonal antibodies (mAbs) directly affect peripheral T cells. Upon anti-CD3 mAb binding the CD3/TCR complex of a naïve T cell, it is capable of shedding or internalizing the receptor/antibody complex or propagating a signaling cascade. This signaling can result in either induction of an anergic program capable of transient immune suppression, or an apoptotic program by the activated cell. Apoptosis causes the release of apoptotic bodies which secrete TGF-β into the environment or are phagocytosed by macrophages which then releases TGF-β into the extracellular space. TGF-β shifts DCs into a tolerogenic state or acts on naïve T cells to induce Tregs. (C) VIP generates tolerogenic DCs from myeloid specific bone marrow progenitors. VIP-differentiated tolerogenic DCs induce T regulatory 1 (Tr1) cells from CD4+ CD25− naïve T cells which secrete anti-inflammatory cytokines such as TGF-β and IL-10. Within an environment associated with factors such as IL-10, TGF-β, and INF-α, VIP facilitates the differentiation of naïve T cells into Tregs with high levels of CTLA-4 on the cell surface. In the presence of VIP, Th3 cells are also induced from the CD4+ CD25− compartment, furthering the anti-inflammatory shift in the microenvironment via the secretion of TGF-β.

We have evaluated the therapeutic potential of granulocyte macrophage-colony stimulating factor (GM-CSF) in animal models of PD, AD, and TBI (Kosloski et al., 2013; Kelso et al., 2015; Gendelman et al., 2017; Kiyota et al., 2018; Schutt et al., 2018) as well as in clinical trials in PD (Gendelman et al., 2017) (NCT03790670) and in AD by others (NCT01409915). GM-CSF is a cytokine known to act as pro-inflammatory and anti-inflammatory modulators, depending on the dose and regimen (Bhattacharya et al., 2015). Anti-inflammatory effects of GM-CSF are due to induction of tolerogenic dendritic cells (DCs) which leads to Treg induction and T cell-mediated tolerance by preventing T cell activation (Fig. 3A) (Bhattacharya et al., 2015; Schutt et al., 2018; Lotfi et al., 2019). GM-CSF promotes the proliferation of both myeloid progenitors in the bone marrow and myeloid lineage cells (Martinez-Moczygemba and Huston, 2003). Bone-marrow derived dendritic cells that differentiate from this expanded progenitor population co-express OX40L and Jagged-1 (Jag-1) which expand natural Tregs and initiate Treg proliferation following interaction with their cognate receptors (OX40 and Notch3) on Treg cells (Bhattacharya et al., 2011; Schutt et al., 2018). Such tolerogenic dendritic cells derived from the bone marrow are also capable of secreting large quantities of TGF-β, which can induce Tregs from Teffs with adequate co-stimulation of the T cell receptor (Lotfi et al., 2019). In addition to mechanisms of Treg induction, GM-CSF promotes the differentiation of CD8a- dendritic cells capable of inducing Tregs from Teffs via antigen presentation (Ganesh et al., 2009). In the MPTP mouse model, GM-CSF administration prior to intoxication or adoptive transfer of GM-CSF-induced Tregs after intoxication attenuated microglial inflammation and protected dopaminergic neurons in the SNc (Kosloski et al., 2013). Furthermore, GM-CSF treatment results in increased frequencies of Tregs and Treg function suggesting that immune modulation with GM-CSF upregulates Treg-mediated immune regulation that is significantly diminished in PD patients and provides an efficacious therapeutic strategy (Saunders et al., 2012; Gendelman et al., 2017).

Clinical studies implementing pharmacological interventions that involve the administration of GM-CSF have been assessed or are ongoing. Recombinant human GM-CSF (rhGM-CSF, sargramostim, Leukine) has been tested in a clinical trial of Crohn’s disease and been shown to increase disease remission (Korzenik et al., 2005). Additionally, the benefits of GM-CSF have been demonstrated in AD models, acute myelogenous leukemia (AML), autologous bone marrow transplantation, and allogeneic bone marrow transplantation (Nemunaitis et al., 1991a; Nemunaitis et al., 1991b; Nemunaitis et al., 1995; Rowe et al., 1995; Kiyota et al., 2018). In a randomized, double-blind, placebo-control phase 1 clinical trial in PD, daily doses of sargramostim or placebo were administered for 8 weeks to PD patients (Gendelman et al., 2017). Sargramostim was safe and well-tolerated, and increased Treg frequencies and function without affecting Teff numbers. Moreover, clinical scores of disease severity were diminished in sargramostim-treated patients and signaling in cortical areas associated with motor function were improved as measured by magnetoencephalography and compared to pre-treatment baselines and placebo-treated controls. Currently, we are evaluating the safety and efficacy of sargramostim dosages and regimen duration in a Phase Ib trial in PD patients (NCT03790670).

In combination with our current clinical trial, we are pursuing additional avenues to enhance the therapeutic potential of GM-CSF. Due to the relatively short half-life of GM-CSF, frequent and high concentration dosing regimens have been required to maintain beneficial plasma concentrations (Cebon et al., 1990; Hovgaard et al., 1992). As a consequence, mild-to-moderate adverse events have been associated with daily administrations including increased WBC counts, injection site reactions, and bone pain (Korzenik et al., 2005; Gendelman et al., 2017). In an effort to diminish these adverse events, we are investigating the effects of a lipid nanoparticle-containing *Csf2* (GM-CSF mRNA) and a long-acting GM-CSF in mouse and rat models of PD. Preliminary data indicate that these formulations increase Treg numbers and Treg function, decrease microgliosis, and increase survival of dopaminergic neurons, suggesting the beneficial utility of long-acting and clearance-protected GM-CSF formulations as potential therapeutic modalities.

Anti-CD3 mAbs have been discovered to provide immunological tolerance by induction of Tregs and Treg function (Fig. 3B). Anti-CD3 mAbs function by binding to the CD3/TCR complex, which effectively eliminates the necessity of activation by cognate antigen such as N-α-syn, and triggers apoptosis or anergy in activated T cells and spares Tregs (Chatenoud et al., 1982; Smith et al., 1997; Penaranda et al., 2011). Concomitant with apoptosis, TGF-β is released into the microenvironment and induces FoxP3 expression in CD4+ T cells, transforming them to express an immunosuppressive functional Treg phenotype. Phagocytosis of the resulting apoptotic bodies by macrophages increases TGF-β levels, further inducing Treg and conferring a tolerogenic phenotype to dendritic cells (Perruche et al., 2008; You et al., 2008). This therapy is currently being tested in clinical trials to treat patients with type I diabetes, inflammatory bowel disease, and chronic hepatitis C infection. While some formulations have shown promising results, others have raised safety concerns that include lymphopenia, cytokine release syndrome, increased rates of infection, and vascular and cardiac issues (Kuhn and Weiner, 2016).

Another promising immunomodulatory agent is the neuropeptide vasoactive intestinal peptide (VIP), a natural hormone that facilitates neuroprotection by increasing Treg number and function, suppressing microglial activation, and reducing neuronal degeneration (Delgado et al., 2005; Fernandez-Martin et al., 2006; Reynolds et al., 2010). Due to its rapid metabolism and clearance as well as dual recognition for binding VIP receptor 1 and 2 (VIPR1 and VIPR2), we developed a selective VIPR2 agonist with increased protease resistance and half-life to address these issues. Treatment with the VIPR2 agonist, LBT36’ resulted in decreased microglial responses and augmented neuroprotection by creating an anti-inflammatory microenvironment, thus altering Th1/Th17 cytokine responses in the MPTP mouse model and in the α-syn overexpression model in rats (Reynolds et al., 2010; Olson et al., 2015; Olson et al., 2016; Mosley et al., 2019).

The influence of VIP is not solely limited to CD4+ CD25+ FoxP3+ Treg induction (Fig. 3C). Tolerogenic dendritic cells differentiate from bone marrow progenitors following exposure to VIP (Chorny et al., 2005; Fernandez-Martin et al., 2006). These dendritic cells are not only capable of facilitating Treg expansion, but also the conversion of naïve T cells to T regulatory 1 (Tr1) cells, further reducing inflammation by means of TGF-β and IL-10 secretion (Varela et al., 2007). VIP also enables the differentiation of Th3 cells from the CD4+ CD25− compartment further augmenting levels of TGF-β within the extracellular environment (Varela et al., 2007). Distinct from anti-CD3 mAbs, VIP is an immunotherapy that doesn’t exclusively target peripheral immune cells, but also biases bone marrow progenitor differentiation towards tolerogenic dendritic cells. Although selective VIP receptor agonists have not yet been tested in clinical cohorts of PD patients, further investigation is merited as VIP has been shown to shift systemic immunity towards a less reactive and more anti-inflammatory phenotype in animal models of PD.

## 7. Concluding remarks

PD and other neurodegenerative disorders and injuries are multi-faceted diseases that involve the CNS and interactions with both adaptive and innate branches of the immune system. In PD, after proteasome-ubiquitin system failure, misfolded α-syn accumulates and initiates an immune cascade that perpetuates neuroinflammation and neurodegeneration. Similarly in AD, fibrillogenic species of Aβ and hyperphoshorylated Tau with increased neurodegeneration of cholinergic neurons, induce neurotoxic microglia with an augmented capacity to drive aberrant Aβ and Tau processing and neuroinflammation. While no specific proteinaceous aberrations have been identified in stroke and TBI, DAMP signals from dead and damaged tissues and neurons initiate astrocytic responses and innate microglial clearance mechanisms. These processes add to the neuroinflammatory environment to facilitate debris clearance and remodeling. The innate pro-inflammatory processes and environment then predisposes the CNS to a wide repertoire of adaptive immune mechanisms. While under conditions of immune homeostasis, the immune system typically favors returning the balance to maintain immune homeostasis. But under conditions whereby immune homeostasis is tipped such that regulatory processes may be compromised, then age, infection, diminished health, and existing proinflammatory conditions may predominate leading to increased injury, inflammation, and neurodegeneration. While current therapies may temporarily subdue some symptoms associated with PD and other neurodegenerative processes, neither neurodegeneration nor disease progression are effectively harnessed. Using immunotherapies, such as GM-CSF, anti-CD3 mAbs, and VIP to modulate the immune system, induce Tregs, and increase their function, neuroinflammation, protein misfolding, and excessive DAMP signaling will ultimately be controlled to better provide neuroprotection and therapeutic outcomes. Future studies to assess the potential translation of these immunomodulatory advancements into other diseases defined by a neurodegenerative disease pathology and chronic neuroinflammation should be prioritized.
